# C-to-U RNA Editing: A Site Directed RNA Editing Tool for Restoration of Genetic Code

**DOI:** 10.3390/genes13091636

**Published:** 2022-09-12

**Authors:** Sonali Bhakta, Toshifumi Tsukahara

**Affiliations:** 1 Bioscience and Biotechnology, School of Advanced Science and Technology, Japan Advanced Institute of Science and Technology (JAIST), 1-1 Asahidai, Nomi City 923-1292, Japan; 2 Department of Anatomy and Histology, Faculty of Veterinary Science, Bangladesh Agricultural University, Mymensingh 2202, Bangladesh

**Keywords:** RNA editing 1, cytidine 2, thymine 3, uridine 4, mutation 5, genetic disease 6

## Abstract

The restoration of genetic code by editing mutated genes is a potential method for the treatment of genetic diseases/disorders. Genetic disorders are caused by the point mutations of thymine (T) to cytidine (C) or guanosine (G) to adenine (A), for which gene editing (editing of mutated genes) is a promising therapeutic technique. In C-to-Uridine (U) RNA editing, it converts the base C-to-U in RNA molecules and leads to nonsynonymous changes when occurring in coding regions; however, for G-to-A mutations, A-to-I editing occurs. Editing of C-to-U is not as physiologically common as that of A-to-I editing. Although hundreds to thousands of coding sites have been found to be C-to-U edited or editable in humans, the biological significance of this phenomenon remains elusive. In this review, we have tried to provide detailed information on physiological and artificial approaches for C-to-U RNA editing.

## 1. Introduction

RNA editing is a biological process or tool for repairing or altering RNA in a mitochondrion-encoded mRNA of a kinetoplastid trypanosome. RNA editing was first introduced to describe a process that occurs in trypanosomes and involves the insertion and deletion of uridine monophosphate (UMP) inside nascent transcripts after transcription [1]. Since the discovery of the post-transcriptional sequence, the number of techniques associated with the term RNA editing has grown. The insertion and deletion of nucleotides other than UMP, base deamination, and the co-transcriptional insertion of non-template nucleotides are now referred to as RNA editing. RNA editing has been observed in mRNAs, tRNAs, and rRNAs, in mitochondrial and chloroplast encoded RNAs, as well as in nuclear encoded RNAs [1]. Examples of RNA editing have been found in many Metazoa, unicellular eukaryotes, such as trypanosomes, and plants. RNA editing has been observed in prokaryotes on a small scale although some researchers have made detailed study on the tRNA editing in *E. coli* [2].

RNA editing tools are basically categorized into two types depending on their response mechanisms. For example, insertion/deletion RNA editing, involves the insertion or deletion of targeted nucleotides with the aim of changing the codon sequence of the targeted mRNA [1]. However, this RNA editing can be done in another way as well, where it turns/alters one encoded nucleotide into a new nucleotide via base alteration or modification without modifying the overall length of the RNA. As a result, the codon sequence is ultimately changed; this is particularly used for the treatment of single nucleotide mutations. In this review, we have tried to focus on the C-to-U editing at RNA level in case of the transcriptomic editing. Thanks to different tools particularly Next generation sequencing (NGS) and Bioinformatics methods, which have developed greatly in the last two decades, it has been possible to detect thousands and thousands of RNA editing events in both plants and mammals [3,4].

## 2. RNA Editing

RNA editing as a therapeutic approach was first conceptualized and utilized as a therapy in 1995. The main purpose of this method is to restore RNA sequences in order to treat genetic diseases caused by point mutations. Advanced research has enhanced and established this technology, which is now known as artificial site-directed RNA editing for restoring RNA. This unique therapeutic approach has the potential to be utilized to cure diseases, such as numerous neurological maladies in humans, by restoring the mutated A or C in mRNA without changing or affecting the genome sequence of the mRNA target [5]. A-to-I and C-to-U editing are two types of substitutional RNA editing in mammals. Due to the higher potential of recoding point mutations, many studies have focused on changing as well as imitating RNA editing. RNA editing of C-to-U is commonly found among flowering plants and mainly occurs within mitochondrial protein regions with highly conserved amino acid sequences [6].

## 3. C-to-U RNA Editing

RNA editing in C-to-U is a process or therapeutic approach that converts a single or multiple C-to-U nucleotides in transcript sequences. C-to-U RNA editing can generate start or stop codons that can change the encoded amino acids depending on preferences towards the splice site [7]. The C-to-U type of RNA editing was originally illustrated in vertebrates for apolipoprotein B (apoB) encoding mRNA. Deamination through hydrolysis at the C4 site of cytidine (C) was later found to be involved in apoB editing [8,9]. The presence of both cis-acting elements (tripartite regulatory sequences) and trans-acting elements around the altered cytidine is required for this conversion or editing (the editosome is a multiprotein complex that contains a catalytic cytidine deaminase and many auxiliary proteins) [5]. Editing of C-to-U at the RNA level has been found in higher family plants, particularly in the mitochondria and chloroplasts [10].

## 4. Artificial C-to-U RNA Editing

Both C-to-U and A-to-I conversions are included in enzymatic site-directed RNA editing. Recently, artificial site-directed RNA editing of A-to-I has been successfully carried out in vitro and in cells as well as in vivo [4,10]. However, few reports of artificial site-directed C-to-U RNA editing have been published recently. The RNA editing machinery relies on two critical components: complementary RNA sequences that can precisely bind to specified sequences (guide RNA) and deamination-editing enzyme/editors. Furthermore, non-enzymatic site-directed C-to-U editing, which does not have the same constraints as site-directed enzymatic RNA editing, was recently identified and has generated a lot of interest in this field of research. In this review, we have focused on C-to-U RNA editing with special emphasis on the enzymatic approach [10].

## 5. Enzymes (Editors) for Artificial C-to-U RNA Editing

**Figure 1:**
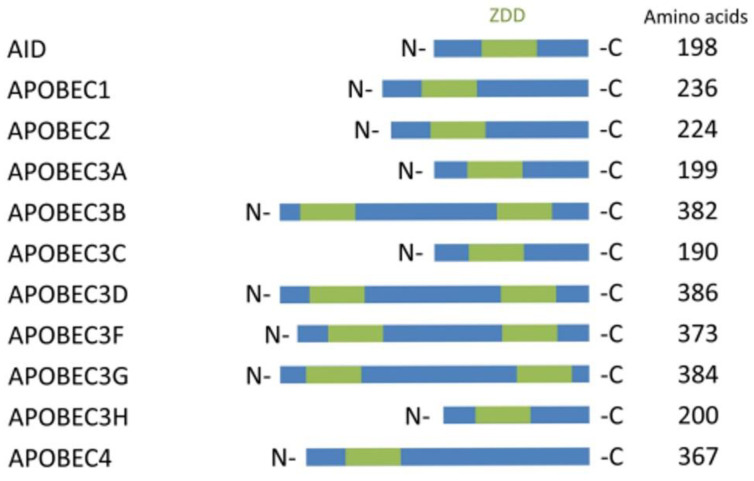
APOBEC family proteins for C to U editing.

The artificial or enzymatic approach of deamination from C-to-U is mainly dependent on the enzymes from the apolipoprotein B mRNA editing catalytic polypeptide-like (APOBEC) family proteins. Eleven genes code for members of the APOBEC family that have been discovered to date (APOBEC1, APOBEC2, APOBEC3A, APOBEC3B, APOBEC3C, APOBEC3D, APOBEC3F, APOBEC3G, APOBEC3H, APOBEC4, and AICDA/AID). They all have a zinc-dependent deaminase domain (ZDD) [11,12]. Among all of these APOBEC subfamily proteins only APOBEC-1, 3A, 3 B, and 3G (Figure 1) have been proven to mediate the C-to-U RNA editing [13,14,15,16]. APOBEC-1 was the first member of the APOBEC family to be discovered and researched, and its significance in apolipoprotein B (ApoB) mRNA editing has been well documented.

**Figure 2:**
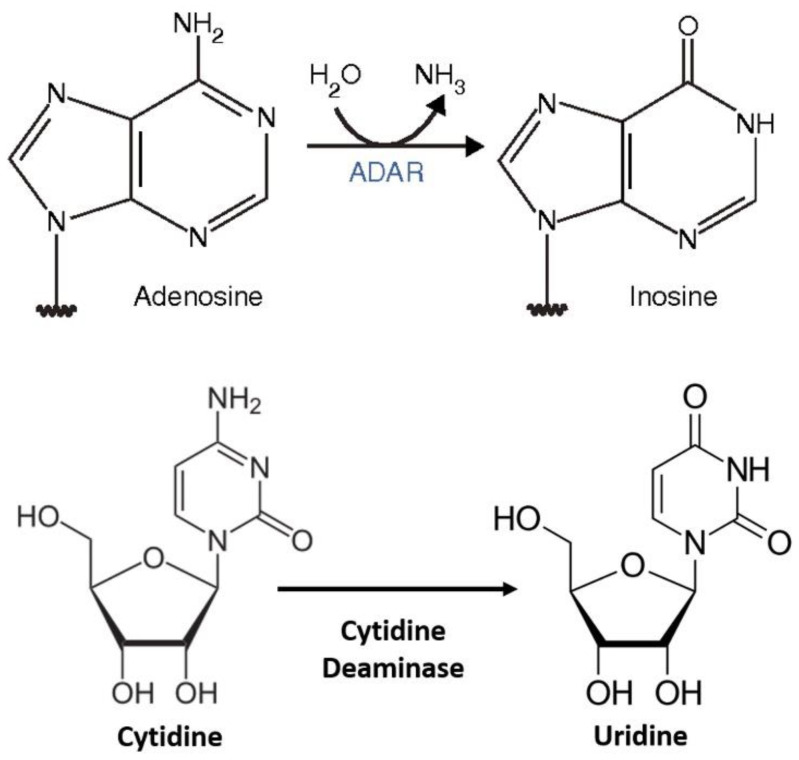
RNA editing (A to I and C to U) [18].

**Figure 3:**
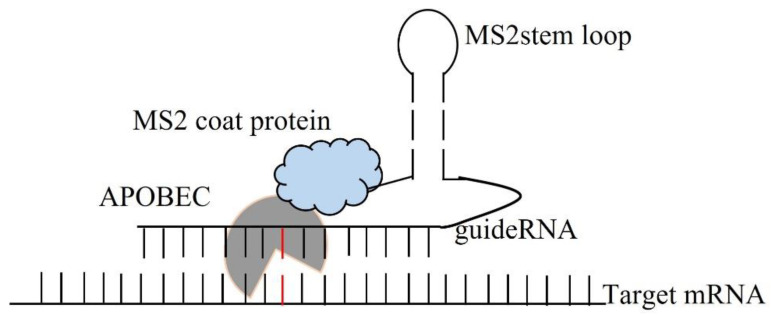
Enzymatic C to U RNA editing by APOBEC 1 deaminase.

For the enzymatic approach, the MS2 system along with the APOBEC family protein enzyme has been a very promising technique for the therapeutic RNA editing technique. C-to-U editing (Figure 2) using the MS2 system (MS2 stem loop along with MS2 coat protein) and APOBEC1 has been previously performed by Bhakta et al. [17] by converting BFP (Blue Fluorescence Protein having a single mutated T-to-C) to GFP (Green Fluorescence Protein- which is restored after the editing from C-to-U) (Figure 3).

Only vertebrates have APOBECs, and this type of RNA editing is the second most common after ADAR (Adenosine Deaminase acting on RNA) editing. Unlike ADARs, the APOBEC family of proteins, including the Alu (Arthrobacter luteus) sequence, primarily affects non-coding and intronic sequences [19,20]. Surprisingly, the APOBEC family of proteins are not just for RNA editing. They were first introduced as tools for the editing of single-stranded DNA (ssDNA) and genomic DNA (gDNA), respectively. As a result, APOBEC-mediated DNA editing has received more attention and is better understood than APOBEC-mediated RNA editing. The efficiency of genome/DNA editing cannot be compared to RNA editing, however, because the deamination of C in DNA results in U; APOBEC-mediated DNA editing can result in the degradation of viral DNA, resulting in a reduction of virus replication or multiplication. Moreover, uracil-rich viral DNA can trigger DNA damage and stress–response pathways, causing natural killer (NK) cells to up-regulate activating ligands (NKG2D ligands) and destroy infected cells [16].

The APOBEC family of proteins plays a vital role in the introduction of mutations in cancerous tissues [21,22,23,24,25]. These mutations are primarily caused by genome/DNA editing or abnormal APOBEC enzyme production. DNA editing mediated by APOBECs for C-to-U editing has been thoroughly characterized by Knisbacher et al. [24], although it is not the basic topic of this current review. DNA editing aids in the natural mechanisms of the body. However, DNA or genome editing is essential for a good and efficient adaptive immune response. Somatic hypermutation is the most commonly known example, occurring in sequences encoding hypervariable portions of immunoglobulins, which result in the formation of high-affinity antibodies [20].

ApoB-100, the full-length form of ApoB protein, is expressed in hepatic cells (hepatocytes) in the liver. APOBEC-1 RNA editing, on the other hand, causes an early stop codon in ApoB mRNA in the small intestine, resulting in the premature termination of translation. Consequently, another isoform of ApoB-48 was created. The full-length form (ApoB-100) carries cholesterol in the bloodstream, whereas the shortened form (ApoB-48) transports triglycerides [26].

There are seven APOBEC-3 paralogs in the human genome (APOBEC-3A, 3 B, 3C, 3D, 3F, 3G, and 3H). Although all of these paralogs bind to RNA [27], only three of them have shown RNA editing activity (APOBEC-3A, APOBEC-3B, and APOBEC-3G). These roles have an impact on the immune system. Under hypoxic conditions and IFN activation, they were found to be expressed in macrophages, monocytes, and NK cells [28,29,30,31]. Furthermore, they are expressed in human natural Tregs in response to CD3/CD28 stimulation, especially APOBEC-3G, 3D, and 3H [31]. Single gene encoded APOBEC-3 was identified for the first time as the Friend leukemia virus resistance (Fvr) gene [32] in mice. Mouse APOBEC3 is most similar to human APOBEC3G and contributes to viral resistance by mutating viral DNA. In recent times the CRISPR-Cas9-APOBEC editing system has had a significant impact on DNA editing, but off-target effects are a major concern in this case [32].

## 6. C-to-U RNA Editing in Mammals

The restoration of genetic code from A-to-I and C-to-U are the two most common RNA editing processes in mammalian cells [33,34]. Deamination has been reported to occur from A-to-I in hundreds of thousands of places, with the most common occurring in intronic and non-coding regions, notably with Alu sequence repeated targets [35,36]. A-to-I RNA editing in coding domains is frequently incorporated with brain proteins that can recode [31]. However, C-to-U editing is less commonly found in humans than is A-to-I [37]. The pre-mRNA of apolipoprotein B (apoB) is predominantly located in intestinal cells, and currently approved targets in mRNA are among the physiologically minimum recognized C-to-U RNA editing targets. In an earlier investigation, previously unknown 32 APOBEC1 (apoB editing catalytic subunit 1) editing sites were identified in the 3′-untranslated regions (3′-UTRs) of diverse mRNA transcripts [38]. Furthermore, in the AU-rich parts of the 3′-UTRs, 56 novel modifying sites were identified, among which 54 were intestinal mRNAs, within which 22 unique editing points were discovered in mRNAs of the liver [39]. In macrophages derived from bone marrow, 410 C-to-U RNA editing events were found, among which 97% of C-to-U events were found to occur in 3′-UTRs [40]. Moreover, C-to-U RNA editing events of apoB pre-mRNA occur in the nucleus [41,42]. At the C6666U editing site, the conversion from glutamine (CAA) to a stop codon (UAA) occurs at the in-frame translational site. The ApoB48 protein is produced by C6666U-edited apoB RNA, whereas ApoB100 protein is produced by C6666-unedited apoB RNA [41].

In the C6802U editing site, the threonine codon (ACA) is changed to an isoleucine (AUA). Because the C6802U editing event occurs concurrently with the C6666U editing event, C6802U is not expressed in the truncated ApoB48 protein but in the mRNA [39]. The RNA editing of C-to-U is necessary for the stoichiometric modulation of trans-acting components within the macromolecular enzyme complex (editosome), which is responsible for targeted deamination. These cis-acting elements, in combination with trans-acting factors, are required for C-to-U RNA editing in vitro. They are made up of 50 nucleotides modifying the edited cytidine that contains a regulatory tripartite motif, which contains an 11-nt motif (UGAUCAGUAUA) located in a sequence (the mooring sequence) downstream of the edited base [43,44,45,46,47]. To generate a stable secondary structure that increases specificity, the 3′ mooring sequence is combined with a 5′ efficiency sequence [45,46].

The editosome of C-to-U editing consists of a minimum of three protein components: ApoB1 and two essential cofactors, ApoB1-complementary factor (ACF) and RNA binding motif 47 (RBM47) [47,48]. The cytidine deaminases (RNA-specific) APOBEC family includes APOBEC1. Like the cytidine deaminase family-derived members, APOBEC1 possesses a zinc-dependent deaminase domain that is essential for the deamination of C [49,50]. In the catalytic domain of APOBEC1, specific amino acids are bound to AU-rich areas in apoB pre-mRNA, producing homodimers. In vitro, this interaction is inadequate for mRNA association, which needs to be used as a cofactor of ACF, a potential RNA-binding protein (RBP). In vitro experiments revealed that this cofactor has a high affinity for the mooring sequence and forms a minimal editosome with APOBEC1 [51]. Elav/HelN1/HuR is a protein that consists of an RNA-recognition motif (RRM) of single-stranded RNA, repeated several times. The N- and C-terminal areas bordering many RRMs are required for the interaction of ACF with the APOBEC1 enzyme [52].

**Figure 4:**
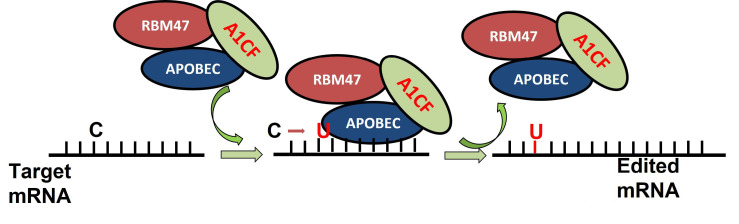
RNA-binding protein RBM47 is required for normal Cytidine to Uridine RNA editing in mammals and is sufficient for the C to U RNA editinf activity of APOBEC demainase domain [52].

While ACF knockout animals may die during early pregnancy, ACF+/mutant mice show a higher editing efficiency, contradicting the idea that cofactors are essential for editosome editability in vitro. Despite the abundance of scientific evidence for cofactors and C-to-U APOBEC-derived deaminase editing in vitro, there is no strong proof that cofactors are essential for C to-U RNA editing in vivo. As a result, the function of cofactors (ACF) in vivo remains unclear [51]. C-to-U RNA editing in vivo requires an extra cofactor, which has recently been discovered as RBM47 [51]. In the holoenzyme of the editosome, RBM47 interacts with APOBEC1 and ACF, and works with APOBEC1 to edit transcripts of ApoB. However, the consequences of the ACF-RBM47 interaction in vivo are not yet fully known. In vitro, RBM47 can also play the role of ACF cofactors in the RNA editing of the C-to-U enzyme complex. The C-to-U RNA editing of apoB and four other C-to-U RNA editing targets (Sult1d1—sulfotransfer RBM47) is an editosome component that is essential for C-to-U RNA editing [53]. A novel enzyme for C-to-U RNA editing has been identified as APOBEC3A (A3A) (Figure 4), DYW, a structurally related member of the cytidine deaminase family, which is expressed mostly in myeloid cells such as macrophages and monocytes [54,55].

## 7. C-to-U RNA Editing in Plants

RNA editing alters the predicted nucleotide sequence in RNA molecules, resulting in a divergence from the genomic sequence of mRNA information for a protein. RNA editing converts C-to-U in the chloroplasts and mitochondria of flowering plants, and converts U-to-C in ferns and mosses. Specific proteins address almost 500 sites of editing in the mitochondria and only 40 sites in plastids of plants with flowering ability, whose genes are increased in plant species with organellar RNA editing [56]. RNA editing randomly restores 400 C-to-U in the mitochondrial mRNA of flowering plants [57].

Although there are significant distinguishing characteristics between mammals and plants C-to-U RNA editing, it may be technically possible to replace the mammalian APOBEC1 enzyme in human therapy applications by combining elements of RNA editing apparatus of plants with guide RNA (a complementary sequence of mRNA that binds with the targeted sequence). However, since C-to-U editing occurs automatically (endogenous property) in plants, the enzymes responsible for the editing/editors can be replaced in plants [58]. The development of this method will be useful in a variety of C-to-U editing situations. As a result, C-to-U editing in plants is represented as an expected correlation, not only parallel to the mammalian process.

In plants, C-to-U and U-to-C are the two commonly found types of RNA editing events. Among them, C-to-U RNA editing is more commonly and precisely found than U-to-C. The RNA editing events of C-to-U occur in both the plastids and mitochondria of plants [58,59,60,61,62].

Higher plant plastids have circular genomes that are 120–130 kb in size, with an expectation of 20–30 cytidines to be converted or altered to uridines, implying a 0.02% editing frequency in the plastid genome [63]. Because there are fewer start codons or stop codons in plastid mRNAs, C-to-U editing allows functional proteins to be generated by the addition of start or stop codons and altering amino acid sequences [64,65,66]. The circular genomes of both organelles encode photosynthesis and respiration genes, respectively. Plastid mRNAs undergo C-to-U RNA editing, but not others, such as transfer RNA (tRNA) or ribosomal RNA (rRNA). Plant mitochondria have a higher editing efficiency of C-to-U RNA editing than plastids; for example, the Arabidopsis thaliana (A. thaliana) mitochondrial genome is approximately 367 kb in size, among which only 30 kb genes are encoded by the respiratory chain complex [67]. However, only 441 C-to-U RNA editing sites were found in mitochondrial ORFs [68]. Unlike plant plastids, mitochondrial C-to-U RNA editing occurs both in mRNA and tRNA, except for rRNA. C-to-U RNA editing is more commonly found in mitochondrial coding sequences than in introns and other UTRs. By restoring and fixing ORFs, these processes aid in gene expression in the mitochondria of plants. Without RNA editing of C-to-U, it would not be possible to produce several respiratory chain proteins. As a result, functionally active mitochondria could not be built in plant cells. Furthermore, by restoring crucial base-pairings, C-to-U RNA editing functionalities in tRNAs of mitochondria are required to restore the folding and processing of tRNA precursors [69,70]. In plants, C-to-U RNA editing requires a variety of editing techniques and approaches. Furthermore, to enhance the specificity of C-to-U editing, nearby cis-elements are required for editing sites. Unlike mammalian cis-elements, several studies have discovered that the majority of recognizing cis-acting elements are found in plants in the editing sites, with the 3′ upstream region contributing only a few percent to C-to-U RNA editing efficiency [71,72,73,74,75]. In most cases, cis-elements containing a 20-nucleotide upstream sequence and a 10-nucleotide downstream sequence are sufficient for RNA editing [76]. When some transacting components are bound to cis-components at the 5′ upstream region, the downstream cytidine is recognized and targeted as an editing site. The RNA editing machinery subsequently moves down towards the editing site, where it is artificially restored to change specific C-to-U.

**Figure 5:**
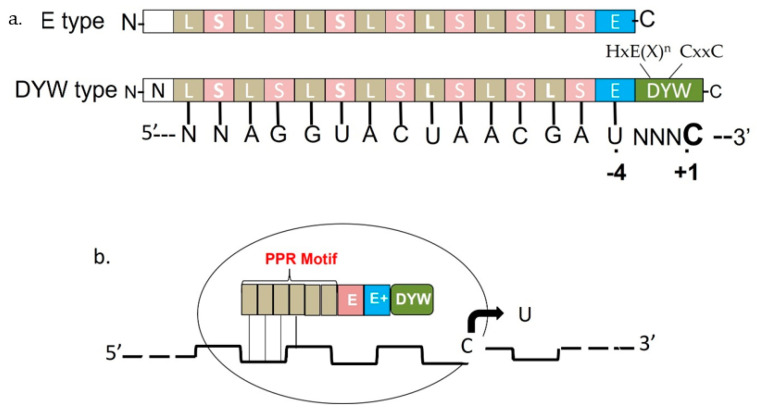
(**a**). Schematic domain structure of PPR editing proteins that consist of PPR motifs (P, L, S), and additional C-terminal domains (E and DYW), (**b**). Involvement of a DYW-type PPR protein in C to U RNA editing [95].

Numerous pentatrico peptide repeat (PPR) motif proteins have been discovered as a part of the trans-acting components required for RNA editing (C-to-U) in both chloroplasts and mitochondria. These PPR motif proteins are the factors responsible for recognizing the site-specificity of cytidines that directly bind to cis-components. PPR motif proteins degenerate 35 repeats of amino acids, numbered from two to thirty [77,78,79]. Based on the PPR motif structure, the PPR family of proteins can be divided into two subfamilies, P and PLS [80]. In *A. thaliana*, there are approximately 450 members of the PPR family, with roughly 250P and 200PLS subfamily members, respectively [79]. PPR motifs are simple in the P subfamily. However, PLS has a triplet of PPR-like motifs, long (L) and short (S), as well as canonical PPR motifs (P). The PLS subfamily can be further divided into E/E+ (Extended) and DYW (Aspartate-tyrosine-tryptophan) classes based on their unique C-terminal domains [80,81] (Figure 5). PLS subfamily members are responsible for trans-acting RNA editing in plants, while P subfamily members are responsible for the process of RNA maturation. The PPR motif is made up of two anti-parallel helices that interact to form a helix-turn-helix motif, which is subsequently connected to a super-helix with a particular central groove by a series of helix-turn-helix motifs. In the central groove, the PPR motif binds to a nucleotide, governing the binding of proteins to specific cis-acting components on the target specific RNA [77,82]. In Arabidopsis, there are almost 650 events of C-to-U RNA editing into the two organelles. Approximately 200 members of the PLS subfamily recognize these sites, and more than two sites can be identified by a single trans-acting factor on average. A minimum of three cis-acting sites in plastids and six points in mitochondria are recognized by CRR22 (chlororespiratory reduction 22) and SOL2, respectively [81,83,84]. RIP or MORFs (RNA-editing factor-interacting protein, also known as multiple site organellar RNA editing factors) [85,86,87,88] and ORRMs (organelle RNA recognition motif factors) have recently been identified as accessory proteins that can cause RNA editing [86,89]. The MORF/RIP family contains 10 A. thaliana members. MORF2/RIP2, MORF9/RIP9, and MORF8/RIP1 have all been found in plastids, with MORF8/RIP1 in mitochondria. The other members of the family are thought to exert their activity in the mitochondria, and RIP10 is encoded by a pseudo-gene. MORF proteins help PPR and other proteins to form spatial connections in an ordered manner, which is necessary for C-to-U RNA editing. MORF proteins may also play a role in the site-specificity of the editing enzyme to the targeted C, which could be linked to their ability to bind metal ions, such as cobalt [85]. ORRM proteins have yet to be discovered, although they may work in a similar way to MORF factors. ORRMs have RRM in the C-terminal region. The ORRM family consists of four essential members, such as ORRM1, which works as a plastid editing factor, whereas ORRM2, ORRM3, and ORRM4 work as mitochondrial RNA editing factors [89,90]. C-to-U substitution editing is thought to act similarly to apoB mRNA editing in plastids and mitochondria, and an APOBEC-1-like cytidine deaminase enzyme may be involved. At least eight cytidine deaminases have been identified in *A. thaliana*. The cytidine deaminase 1 (At-CDA-1) protein from *A. thaliana* has been discovered; however, it does not show any RNA editing capacity [91]. These findings indicate that a nuclear-encoded protein that has yet to be identified is exported to these organelles, and the components of RNA editing in plant mechanisms remain unknown. RNA editing is mostly found in the seedlings and leaves of A. thaliana. In 12-day-old seedlings and the leaves of 21-day-old seedlings, substantial U-to-C and A-to-I (G) RNA editing events have recently been described [92]. Furthermore, U-to-C conversion was found to be the most common RNA editing event in mature mRNA untranslated regions (UTRs) of mature mRNA, followed by uridine to guanine (U-to-G) editing [93]. Moreover, a previous study using RNA sequencing of 12- and 20-day old *A. thaliana* seedlings identified specific U-to-C RNA editing events [92,94], prompting us to investigate the implication of the genes in RNA editing events of U-to-C in *A. thaliana*.

At the positions of translation of nuclear transcripts, AT1G29930.1 and AT1G52400.1, the RNA editing events of C-to-U and U-to-C are both seen in A. thaliana [96]. Because of the deamination of C-to-U and amination of U-to-C, reactions are also visualized at neighboring locations. Although amination occurs more frequently than deamination, the reaction of deamination operates as the amino group donor for the amination reaction [96]. Despite this, the authors were unable to identify similar editing events in the RNA-seq data. As a result, in plants, the amino group donor of U-to-C amination is unknown. However, in the cDNA of AT3G47965, a small T was superposed with C, slightly upstream of the edited T, indicating a putative donor of the amino group. Previously, the parallel analysis of RNA ends (PARE) and massive parallel signature sequencing (MPSS) data was used to study editing sites in nuclear transcripts for mRNA. Although it was discovered that the nuclear genes may contain all 12 RNA editing patterns, the number of editing sites may vary in different patterns. According to these findings, RNA editing is an important RNA-based regulatory technique for nuclear genes, as well as mitochondrial and chloroplast genes. However, a comprehensive concept of RNA editing in nuclear protein-coding transcripts in plants is yet to be achieved [96,97]. However, the DYW domain was recently isolated from A. thaliana (unpublished) and utilized in conjunction with the MS2 system to restore the genetic code from mutated C-to-edited/restored U (BFP to GFP) by following the principle proposed by Bhakta et al., 2020, which had previously been employed for A-to-I editing with MS2-ADAR1 editase [98,99,100].

## 8. Human Diseases Related to C-to-U Editing

While it is evident that C-to-U RNA editing occurs frequently, because APOBEC1 expression in humans is thought to be tissue-specific, a majority of the illness must be assigned to RNA editors of A3, which have yet to be studied fully. Despite the fact that all the members of the AID or APOBEC family of proteins were originally classified as DNA mutators or RNA editors, structural data (all active sites appear to be nearly identical; reviewed in [101]) and cell biology observations have yet to clarify the classification (APOBEC1 has the functionality of a robust DNA editor) [102,103]. There are different diseases caused by the T to C mutations in humans (Table 1) which could be treated with the enzymatic approaches.

It has been kept in mind that when considering the impact of RNA editing in certain illnesses, most of the deaminases may be used for both DNA and RNA editing. Although APOBEC1 is now thought to be expressed in the outer part of the human digestive system, surprisingly, all the genetic illnesses associated with it have recently been found in the human brain. GlyR modification is linked to the onset of temporal lobe epilepsy (TLE) [102]. In the hippocampus part of the brain with pharmaco-resistant TLE, GlyR editing was found to be higher [104].

The terminals of presynaptic regions of hippocampal neurons contain RNA-edited GlyRs, and even slight modifications in editing can cause malfunction [105,106]. APOBEC1 may also modify GlyR mRNA in vitro, which was discovered at the same time. There are two genetic variations in APOBEC1: 80M and 80I. Recent bioinformatics studies have found that they have been linked to GlyR editing levels [107]. Patients with intractable TLE (iTLE) were also tested for dimorphism of APOBEC1, which revealed that the patients with the 80I variant had simple or complex seizures, while those with 80M had neurodegenerative and generalized seizure action [107]. However, the 80M polymorphism in human APOBEC1 has previously been shown to have no considerable impact on APOBEC-mediated RNA editing in the small intestine [108]. The A3 family, as well as entities of disease in which APOBEC1 and A3 are applied, must be investigated in the same way. In the nervous system, alternative splicing can occur at the transcript encoding tryptophan hydroxylase 2 (TPH2a) from editing of C-to-U. Exon 3b undergoes C-to-U editing, which results in a mutation (Q129X substitution) and a shorter protein. Editing at this position significantly decreases in suicide-prone and schizophrenia patients (by 50% and 30%, respectively) [109]. Mutations in APOBEC1 cofactors have also been linked to this disease. RBM47 mutations have been associated with the growth of breast cancer, specifically an increase in the fitness of select cancer clones and a higher metastatic potential [110]. RBM47 expression has also been linked to a better prognosis in individuals with lung, breast, and gastrointestinal cancer growth, indicating a tumor-suppressive effect [111]. Although these studies did not delve into mRNA editing, RBM47 is known to bind to approximately 2500 transcripts. It is crucial to determine whether, in some transcripts, the loss of RNA editing function can be caused by the loss of the editing co-factor and, finally, can be linked to disease progression. Some RBM47-bound RNA targets [112] have also been identified as targets for APOBEC1-mediated RNA editing or APOBEC1-mediated RNA interactors, such as microglobulin-2 (B2m) and interleukin-8 (IL8) [113]. In A3 RNA editors, these polymorphisms may affect the progression of human disease by affecting immune responses or RNA editing levels or targets. Patients with systemic lupus erythematosus (SLE) have high levels of circulating type I interferon and increased expression of interferon-stimulated genes, including various A3s [114,115]. SLE patients have also been found to have higher levels of A-to-I (through adenosine deaminases) and C-to-U RNA editing [116]. Amino acid recoding and the creation of MHC class I epitopes can both contribute to the progression of illness as a result of this elevated amount of editing [111]. The editing of A3A and A3RNA G appears to be more careful than that of their DNA [117], which has a more specific sequence signature.

RNA editing was more likely to be observed in stem-loop structures with target C included in the stem loop, wherein the levels of RNA editing are proportional to the stability of the stem loop structure [112]. However, DNA editing mediated by A3A and A3G appeared to be non-specific, with dinucleotide [T/C]C as the preferred target only. The rs172378 C1q synonymous SNP, which has been linked to nephritis in SLE patients [116,117,118,119], has increased C-to-U editing, most likely by altering the RNA secondary structure and stabilizing a stem-loop [120]. This type of SNP affects the way the A3s target RNA. While it is unknown whether this mutation causes SLE directly, it is possible that, similar to other RNA editing events, it influences transcript fate and, as a result, protein output, contributing to the disease. Similarly, additional SNPs, including synonymous ones, can alter protein diversity by causing changes in RNA editing. This type of SNP affects the way the A3s target RNA. While it is unknown whether this mutation causes SLE directly, it is possible that, like other RNA editing events, it influences transcript fate and, as a result, protein output, contributing to the disease. Similarly, additional SNPs, including synonymous ones, can alter protein diversity by causing changes in RNA editing.

## 9. Future Perspectives of RNA Editing in Diagnosis and Treatment

According to the findings presented in this review, RNA editing levels, as well as the expression of A and C deaminases and specifically altered genes (tumor suppressors and oncogenes), could be important predictors of cancer pathogenesis and progression [121,122,123]. Non-regulated patterns of ADAR and APOBEC expression in tumorous and healthy tissues revealed a promising clinical strategy for an improved means of cancer detection and therapy [124,125,126]. Genes that have been altered in a particular way play a vital role in tumor pathophysiology [127,128,129]. Non-synonymous events of RNA editing and expression levels, which have major applications in drug sensitivity, such as tamoxifen resistance in ER2+ breast malignancies [130,131], also represent a barrier to therapeutic choices. Furthermore, these processes provide new targets for therapeutic approaches. ADAR inhibitors are a novel and effective therapy for ADAR-overexpressing malignancies, such as breast and lung cancers [132]. Moreover, due to the recent functionality of APOBEC inhibitors in cancer, they are still in the early stages of development, which is of great interest in this field [133]. RNA editing, as well as its regulation and application, offers many possibilities. In addition to traditional deaminase inhibitors for regulating expression, molecular methods, such as antisense oligonucleotides, are potent and selective inhibitors of RNA editing on targeted RNAs [134]. An artificial guide mediated RNA editing approach [18,135,136,137,138,139] is a technique that can be utilized for the treatment of hypo-edited diseases such as prostate and brain tumors, as well as disease-promoting genetic changes.

## 10. Conclusions

Although initial evidence on RNA editing events in humans was first published in the late 1980s, in respect of hepatocytes of the liver (C-to-U editing in ApoB mRNA was published in 1987), this topic has only recently received further attention. The role of RNA editing in the initiation, progression, and metastasis of cancer has been extensively researched in a range of cancer types, with a growing body of evidence pointing in that direction. However, many concerns remain unanswered, and the importance of RNA editing processes in cancer and other human diseases remains unclear. Furthermore, substantial RNA editing in transcripts encoding cancer-related proteins increases the likelihood of neoantigen formation. More research into RNA editing could lead to advancements in cancer immunotherapy and/or targeted anti-cancer drugs, as lymphocytes can swiftly recognize such neoantigens.

## Figures and Tables

**Table 1:** Diseases caused by T to C mutations.

No.	Disease State	Gene Symbol	Base Change	Amino Acid	Codon
1	ADA deficiency	ADA	CTG-CCG	Leu-Pro	107
2	APRT Deficiency	ART	ATG-ACG	Met-Thr	136
3	Amyloid prealbumin	PALB	GTG-GCG	Val-Ala	30
4	Antithrombin III def.	AT3	TTC-TCC	Phe-Ser	402
5	Antitrypsin ∝ 1 def.	PI	CTC-CCG	Leu-Pro	41
6	Antitrypsin ∝1 def.	PI	CTC-GCG	Val-Ala	213
7	Elliptocytosis	SPTA	CTC-CCG	Leu-Pro	207
8	Epidermolysis bull	KRT14	CTG-CCG	Leu-Pro	384
9	G6PD Deficiency	G6PD	CTG-CCG	Leu-pro	968
10	Galactosaemia	GALT	CTG-CCG	Leu-Pro	195
11	Gangliosidosis GM1	GLB1	ATC-ACC	Ile-Thr	51
12	HPRT deficiency	HPRT	ATT-ACT	Ile-Thr	182
13	Haemolytic anaemia	PGK	CTG-CCG	Leu-Pro	88
14	Haemophilia A	F8	TTC-TCC	Phe-Ser	293
15	Haemophilia A	F8	TTG-TCG	Leu-Ser	2166
16	Insulin Resistance	INSR	CTG-CCG	Leu-Pro	233
17	Laron dwarfism	GHR	TTT-TCT	Phe-Ser	96
18	Leukocyte adhes. Def.	LFA1	CTA-CCA	Leu-Pro	149
19	Lipoprt. lipase def.	LPL	ATT-ACT	Ile-Thr	194
20	MCAD deficiency	MCAD	ATA-ACA	Ile-thr	375
21	Methaemoglobin	DIA1	CTG-CCG	Leu-Pro	148
22	Neurofibromatos is (1)	NF1	CTC-CCG	Leu-Pro	
23	OTC deficiency	OTC	CTA-CCA	Leu-Pro	45
24	OTC deficiency	OTC	CTT-CCT	Leu Pro	111
25	Phenylketonuria	PAH	TTG-TCG	Leu-Ser	48
26	Phenylketonuria	PAH	TTG-TCG	Leu-Ser	255
27	Phenylketonuria	PAH	CTG-CCG	Leu-Pro	311
28	Pompe disease	GAA	ATG-ACG	Met-Thr	318
29	Retinitis pigmentosa	RDS	CTG-CCG	Leu-Pro	185
30	Ster.18-hydrox. Def.	CYP18	GTG-GCG	Val-Ala	386
31	Thalassaemia ∝	HBA2	ATG-ACG	Met-Thr	−1
32	Thalassaemia ∝	HBA2	CTG-CCG	Leu-Pro	125
33	Thalassaemia ∝	HBB	CTG-CCG	Leu-Pro	110
34	Thalassaemia ∝	HBD	CTG-CCG	Leu-Pro	141

## Data Availability

Not applicable.
